# Effect of indirect composite treatment microtensile bond strength of self-adhesive resin cements

**DOI:** 10.4317/jced.52754

**Published:** 2016-02-01

**Authors:** María-Victoria Fuentes, Nuria Escribano, Bruno Baracco, Martin Romero, Laura Ceballos

**Affiliations:** 1 Assistant Professor, Department of Stomatology and Nursing, Health Sciences Faculty, Rey Juan Carlos University, Alcorcón, Spain; 2 Associate Professor, Department of Stomatology and Nursing, Health Sciences Faculty, Rey Juan Carlos University, Alcorcón, Spain

## Abstract

**Background:**

No specific indications about the pre-treatment of indirect composite restorations is provided by the manufacturers of most self-adhesive resin cements. The potential effect of silane treatment to the bond strength of the complete tooth/indirect restoration complex is not available.The aim of this study was to determine the contribution of different surface treatments on microtensile bond strength of composite overlays to dentin using several self-adhesive resin cements and a total-etch one.

**Material and Methods:**

Composite overlays were fabricated and bonding surfaces were airborne-particle abraded and randomly assigned to two different surface treatments: no treatment or silane application (RelyX Ceramic Primer) followed by an adhesive (Adper Scotchbond 1 XT). Composite overlays were luted to flat dentin surfaces using the following self-adhesive resin cements: RelyX Unicem, G-Cem, Speedcem, Maxcem Elite or Smartcem2, and the total-etch resin cement RelyX ARC. After 24 h, bonded specimens were cut into sticks 1 mm thick and stressed in tension until failure. Two-way ANOVA and SNK tests were applied at α=0.05.

**Results:**

Bond strength values were significantly influenced by the resin cement used (*p*<0.001). However, composite surface treatment and the interaction between the resin cement applied and surface treatment did not significantly affect dentin bond strength (*p*>0.05). All self-adhesive resin cements showed lower bond strength values than the total-etch RelyX ARC. Among self-adhesive resin cements, RelyX Unicem and G-Cem attained statistically higher bond strength values. Smartcem2 and Maxcem Elite exhibited 80-90% of pre-test failures.

**Conclusions:**

The silane and adhesive application after indirect resin composite sandblasting did not improve the bond strength of dentin-composite overlay complex. Selection of the resin cement seems to be a more relevant factor when bonding indirect composites to dentin than its surface treatment.

** Key words:**Bond strength, self-adhesive cement, silane, dentin, indirect composite.

## Introduction

Indirect resin composite restorations are now routinely used to restore teeth with extensive coronal loss as they are more resistant to occlusal wear than direct composite restorations and allow achieving proper proximal contacts and occlusal anatomy (1). Moreover, they show several advantages over ceramic inlays or onlays as they cause less wear of the opposing dentition, are easier to finish, polish and repair (1).

The long-term clinical success of these restorations is largely determined by the bonding effectiveness of the resin’s luting agent to tooth substrate and to processed resin composite (2), as indirect procedures double the adhesive interfaces (3).

Adhesion of resin cement to processed composite restorations is challenging. Indirect composite restorations are subjected to an additional post-cure under pressure, vacuum, inert gas, light, heat or a combination of these conditions, to increase resin conversion and enhance their physical properties, but it lessens the potential for chemical bonding as the quantity of residual free carbon double bonds decreases (1,4). Several treatments of composite fit surfaces have been proposed in order to improve either micro-mechanical retention or chemical bonding. The combination of sandblasting of the intaglio surfaces, followed by silanization has been proposed in order to improve micro-mechanical retention and chemical bonding, respectively (5). However, there is no consensus about the influence of intermediary agents (silane coupling agent and dentin adhesive) on adhesive properties of composite restorations (4).

Silane coupling agents are bifunctional molecules used to induce a chemical bond between the inorganic fillers of the indirect composite and the methacrylate monomers of the resin cement matrix (2). Moreover, silane agents increase the wettability of the composite by making the surface hydrophobic (4). The subsequent application of resin adhesive as an intermediate agent would facilitate wetting of the resin cement to the aluminum oxide air-abraded composite (6) and most researchers consider it to be essential for higher composite repair strength (7).

On the other hand, adhesion to tooth structure is accomplished by the application of dual-cure resin cements that may require the application of an etch-and-rinse adhesive system, a self-etching primer or no tooth pre-treatment as occurs with self-adhesive resin cements (8). This simplicity in the clinical procedure together with their ability to bond to different restorative substrates has made these self-adhesive luting agents increasingly popular (8).

However, no specific indication about the pre-treatment of indirect composite restorations is provided by the manufacturers of self-adhesive resin cements. Only for RelyX Unicem, sandblasting with aluminium oxide particles is recommended.

Therefore, the purpose of this in vitro study was to determine the contribution of silane and an adhesive application after sandblasting of the intaglio surfaces of indirect composite restorations on microtensile bond strength to dentin of five self-adhesive and one total-etch resin cements. The null hypotheses were that strength values are not influenced by silane and adhesive application on the indirect composite surface and that self-adhesive resin cements provide similar bond strengths to dentin as a total-etch resin cement.

## Material and Methods

-Tooth preparation

Intact caries-free extracted human third molars were selected for this study. All teeth were stored in a thymol solution at 4°C until their use. Flat coronal dentin surfaces were exposed by removing occlusal enamel and superficial dentin with a slow-speed, water-cooled diamond saw (Isomet 5000, Buehler, Lake Bluff, IL, USA). Dentin surfaces were abraded with wet 500 grit SiC papers to create standardized smear layers. Prior to the luting procedure, dentin surfaces were copiously rinsed with water and blot-dried with absorbent paper for 5 s.

-Composite overlays preparation

Composite cylinders were prepared by applying incremental layers 1.5-mm thick of a microhybrid resin composite (Filtek Z250, A3 shade; 3M ESPE, St. Paul, MN, USA) into a teflon mold (8 mm in diameter and 3 mm high). Each increment was light-cured for 40 s with a LED unit (Bluephase, Ivoclar Vivadent, Schaan, Liechtenstein) using the high intensity program (1200 mW/cm2). Afterwards, the composite blocks were polymerized in an oven (Lumamat 100; Ivoclar Vivadent, Schaan, Liechtenstein) with program 3, at 104°C and high light intensity for 25 min.

The bonding surface of each composite restoration was airborne-particle abraded with 50 µm aluminum oxide particles (Rondo-flex 2013; Kavo, Biberach, Germany) for 10 s at a distance of 10 mm, perpendicularly to the surface. Before luting procedures, the composite blocks were also ultrasonically cleaned for 5 min in distilled water and air-dried.

Half of the composite cylinders did not receive additional chemical surface treatment (Treatment NOT). In the remaining composite restorations (Treatment SA), the intaglio surfaces were additionally primed with a silane coupling agent (RelyX Ceramic Primer, 3M ESPE, St. Paul, MN, USA) and an adhesive (Adper Scotchbond 1 XT, 3M ESPE, St. Paul, MN, USA). The silane agent was applied and allowed to evaporate for 60 s and then further air dried for 15 s. Afterwards, a thin layer of adhesive was applied on the silanized surface and light-cured for 20 s with the Bluephase unit.

-Indirect composite overlays bonding

Teeth were randomly distributed into 12 experimental groups according to the surface treatment applied on the composite over-lays and to the resin cement used.

Five self-adhesive resin cements were investigated, RelyX Unicem (3M ESPE, St Paul, MN, USA), G-Cem (GC corp., Tokyo, Japan), Speedcem (Ivoclar Vivadent, Schaan, Liechtenstein), Maxcem Elite (KerrHawe, Orange, CA, USA) and Smartcem2 (Dentsply, Konstanz, Germany) in comparison with the total-etch resin cement, RelyX ARC (3M ESPE, St. Paul, MN, USA).

The resin cements were mixed and applied according to the manufacturers’ instructions listed in  Table 1. Each composite cylinder was luted to the dentin substrate under a constant pressure of 1 Kg during the first 5 min, leaving the material to set in the self-curing modality. Finally, curing was completed by light irradiation from the top of the 3 mm thick composite cylinder for 80 s with a LED curing unit (Bluephase, output: 1200 mW/cm2). The bonded specimens were stored in a laboratory oven for 24 h at 37°C and 100% relative humidity until the microtensile bond strength test was performed.

**Table 1:**
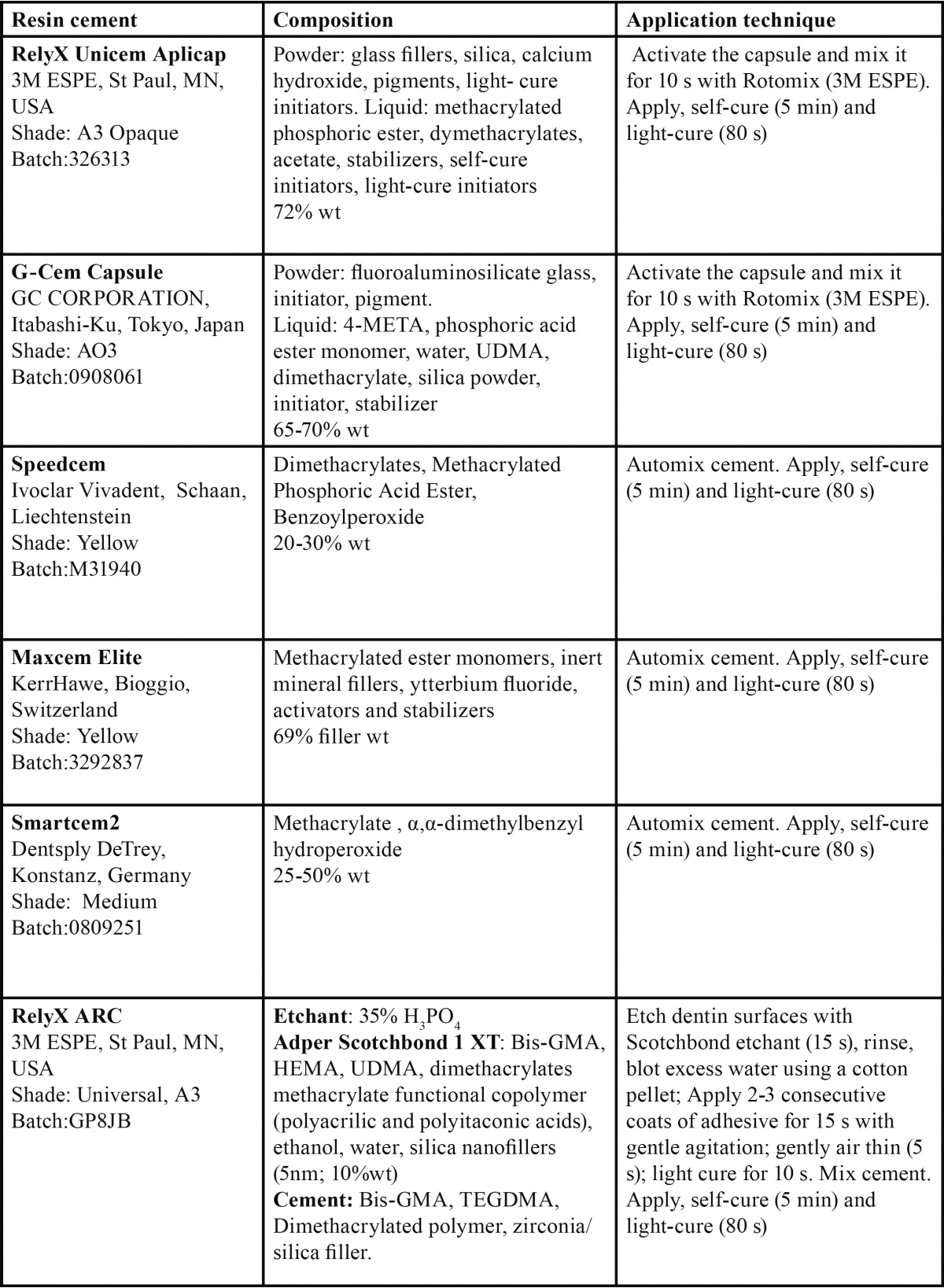
Composition and application technique of the resin cements tested according to information provided by manufacturers.

-Microtensile bond strength test

After the storage period, the bonded assemblies were sectioned with a water-cooled diamond saw (Isomet 5000, Buehler, Lake Bluff, IL, USA) in both, x and y directions, perpendicularly to the adhesive interface to obtain sticks with a cross-sectional area of approximately 1 mm2. All sticks were measured using a digital caliper (Mitutoyo Corp, Kanogawa, Japan). Specimens were attached to the fixtures of a universal testing machine (Instron 3345, Instron Co, Canton, MA, USA) with a cyanocrylate adhesive (Loctite Super Glue-3 gel; Henkel, Düsseldorf, Germany) and stressed to failure in tension at a cross-head speed of 1 mm/min. The bond strength values were calculated in MPa.

Failure modes were evaluated under a stereomicroscope (Olympus SZX7, Olympus Co., Tokyo, Japan) at 40x magnification and classified as cohesive (within the cement, dentin or composite), adhesive (between cement/dentin, or composite/cement, or both cement/dentin and composite/cement fractures) or mixed (adhesive and cohesive fractures occurred simultaneously). Each type of failure mode was expressed as a percentage of the total number of specimens. Characteristic de-bonded specimens, with microtensile bond strength values and failure patterns similar to those most frequently detected in each experimental group, were sputter-coated with gold (SCD 005 Sputter Coater, BalTec, Balzers, Liechtenstein) and observed under scanning electron microscopy at 20 kV (SEM; Hitachi VP-SEM S-3400N, Tokyo, Japan).

-Statistical analysis

A two-way ANOVA was applied to analyse the effect of composite surface treatment and resin cement used on microtensile bond strength. Post-hoc comparisons were performed by Student-Newman-Keuls test. Statistical analysis was carried out without considering pre-test failures. All tests were performed at a pre-set alpha of 0.05 by means of IBM SPSS 18 (IBM Company, Chicago, IL, USA).

## Results

Descriptive statistics and percentage of pre-test failures for each experimental group are summarized in  Table 2, along with statistical significance of the differences. Maxcem Elite and Smartcem2 bond strength results were removed from the statistical analysis due to the high pre-test failures incidence, 86.4% and 88.1%, respectively.

**Table 2:**
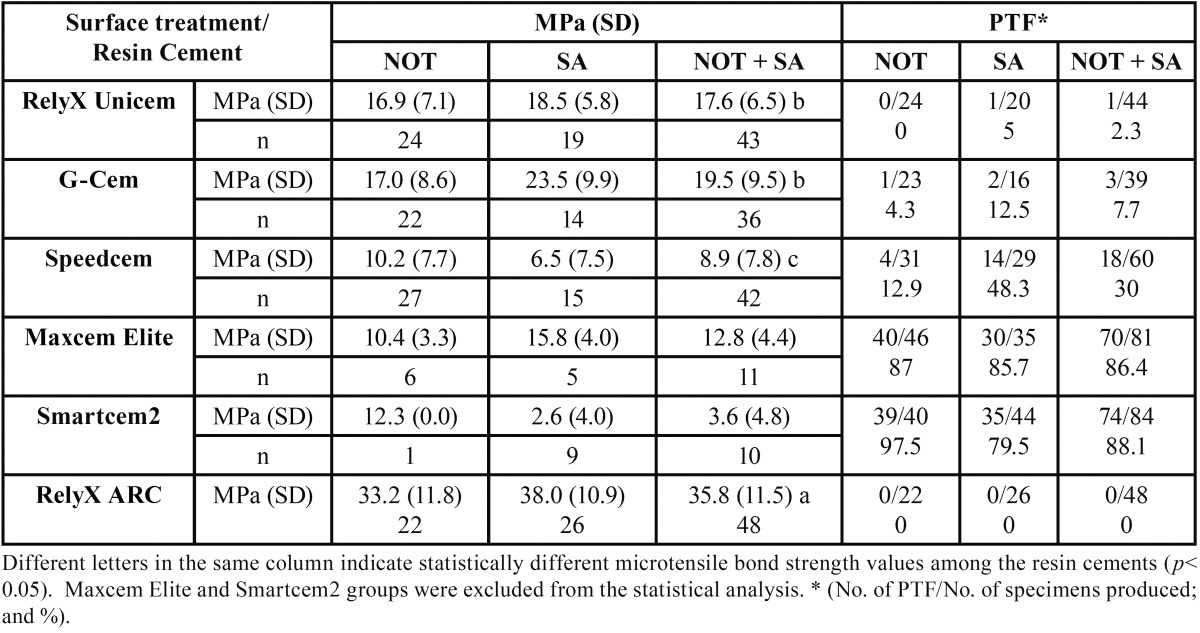
Means microtensile bond strength in MPa (standard deviation) and number and percentage % of pre-test failures (PTF) of resin cements to different treated indirect composites.

Bond strength values were significantly influenced by the resin cement used (F=70.328, *p*<0.001). However, composite surface treatment (F=2.718, *p*=0.101) and the interaction between the resin cement applied and surface treatment (F=2.473, *p*=0.06) did not significantly affect dentin bond strength. According to this, differences among resin cements were analysed regardless of indirect composite resin treatment.

The total-etch resin cement RelyX ARC displayed the highest microtensile bond strength mean values, without pre-test failures incidence. All self-adhesive resin cements showed lower bond strength values than RelyX ARC. Among self-adhesive resin cements, RelyX Unicem and G-Cem attained statistically higher bond strength values than Speedcem.

**Figure 1:**
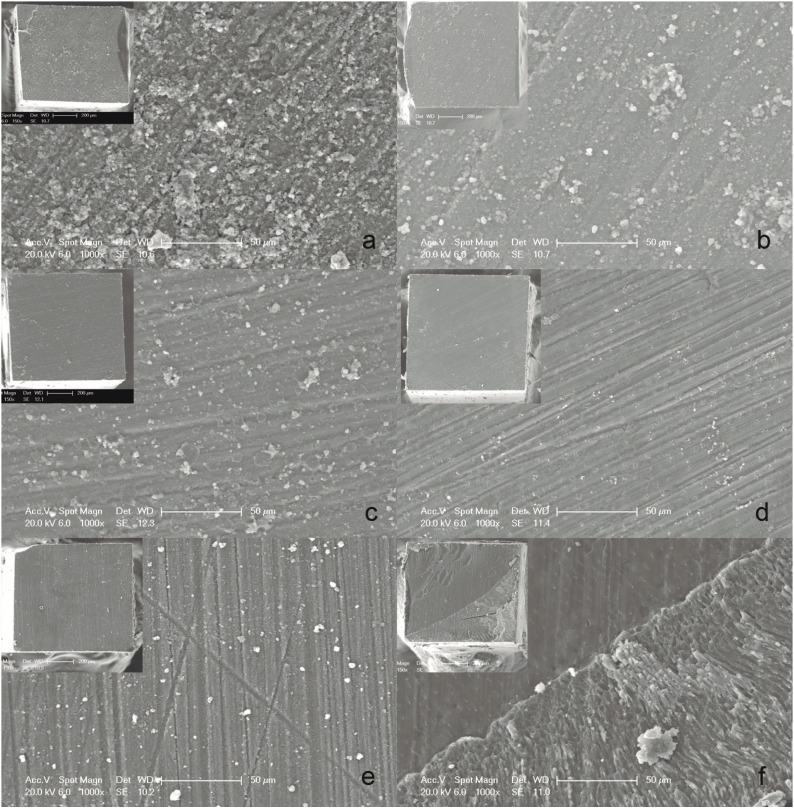
a-e) show adhesive fracture pattern between dentin and indirect restorations when self-adhesive resin cements were used. (a:RelyX Unicem, b: G-Cem, c: Speedcem, d: Maxcem Elite, e: Smartcem2), exhibiting a complete detachment of the self-adhesive resin cements tested from the dentin substrate. f) shows a mixed failure on a dentin surface from a specimen luted with RelyX ARC.

The distribution of failure modes observed is shown in  Table 3 and representative SEM micrographs of fractured beams are displayed in figure 1. For all self-adhesive resin cements tested, the predominant failure mode was adhesive, between the resin cement and dentin. Regarding RelyX ARC, the distribution of the fracture modes was heterogeneous, as it showed more adhesive failures between cement and composite and mixed fractures than the self-adhesive resin cements did.

**Table 3:**
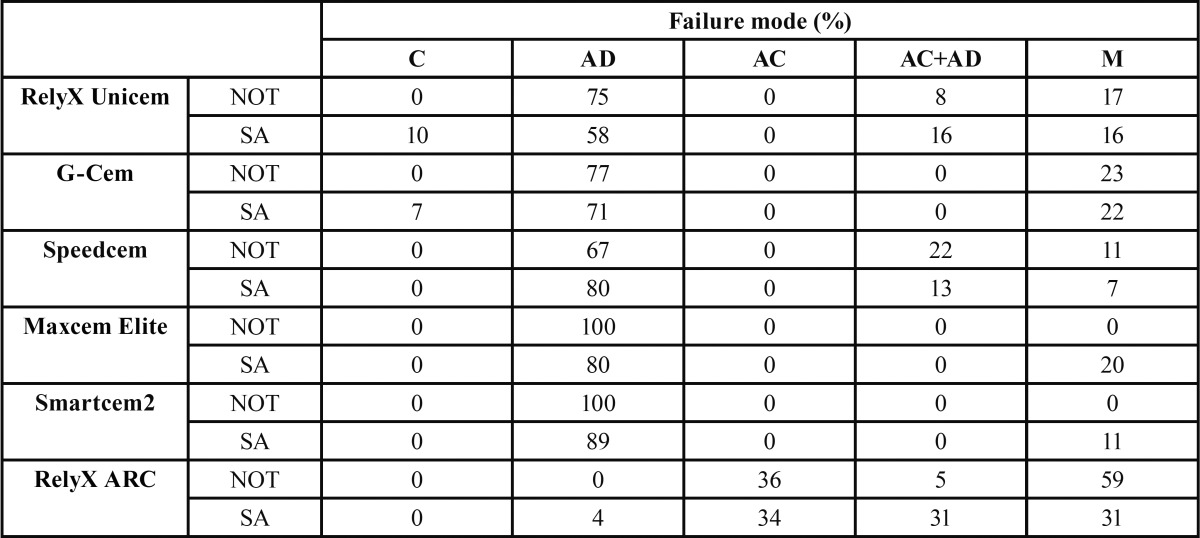
Failure mode distribution (%). Abbreviation: C, cohesive (within the cement, dentin or composite); AD, adhesive between the cement and dentin; AC, adhesive between the composite and the cement; AD+AC, adhesive at the dentin/cement level and composite/cement simultaneously; M, mixed.

## Discussion

According to the obtained results, resin cements tested do not require application of intermediary agents (silane plus adhesive) to micro-retentive Filtek Z250 overlays to enhance the bonding capacity of dentin-indirect composite complex. Therefore, the first null hypothesis must be accepted. On the contrary, the second hypothesis must be rejected, as the total-etch resin cement provided statistically higher bond strength values than the self-adhesive resin cements tested. Therefore, selection of the resin cement seems to be a more relevant factor when bonding indirect composites to dentin than surface treatment.

In this study, all indirect composite surfaces were previously sandblasted with aluminum particles. This treatment roughens the composite, removes some of the resin matrix, and leaves exposed filler particles on the surface (9), promoting micromechanical retention between different components. The efficacy of air abrasion treatments depends on the type of resin composite (10). In agreement with our results, it has been previously reported that treatments based in sandblasting, aluminum oxide or silica coating, produce higher mean bond strength values of repaired and aged Filtek Z250, independently of the intermediary agent applied (silane, adhesive or the association of both) (10). It should be highlighted that although silica coating enables the chemical interaction with silane, no significant increase in mean bond strength values was detected in comparison with aluminum oxide sandblast (10). Therefore, it seems that sandblasting treatment is the main responsible factor for improving the retentive properties on indirect composite restorations (4). In the present study, the predominant failure of self-adhesive resin cements was adhesive between resin cement and dentin. Only the conventional multi-step resin cement ARC showed some variation in fracture mode, with a significant number of mixed and adhesive failures. The present findings (no influence of pretreatment of indirect composite) confirm those previously reported (11) using dentin as the substrate for adhesion. However, Dos Santos *et al.* (12) found that pretreatment with the combined used of silane and adhesive, improved the bonding potential of self-adhesive resin cements. Unlike our study, these authors tested the bonding ability of RelyX Unicem or BisCem (Bisco Inc., Schaumburg, IL, USA) to a composite restoration and not dentin.

The resin cement RelyX ARC yielded significantly higher bond strength values than the self-adhesive cements tested. Previous papers have also reported a relatively reduced bonding effectiveness of self-adhesive resin cements when compared with other total-etch resin cements (13). Regarding RelyX ARC, recent papers also have shown a higher bonding ability than self-adhesive resin cements (11,14). Only in a recently published study (15), RelyX Unicem obtained higher bond strength values. This superiority of the conventional resin cement has been attributed to the etch-and-rinse adhesive applied, Adper Scotchbond 1 XT, with RelyX ARC. The phosphoric acid, required in the total-etch technique, is able to completely remove the smear layer and smear plugs and to demineralize the underlying dentin, allowing the infiltration of the collagen network by the adhesive resin and the establishment of multiple resin tags and a thick hybrid layer, thus providing micromechanical retention (14). Regarding the self-adhesive resin cements tested, the results confirm that self-adhesive resin cements are a heterogeneous group of materials (16). They possess a complex composition with presence of conventional mono-, di-, and/or multi-methacrylate monomers, phosphate and phosphonate acid-functionalized monomers, fillers, and a redox and a photoinitiator (8). The selection and concentration of each component is relevant for the final performance of these materials. Specifically, acidic monomers must be strong enough to produce an adequate etching of dentin with stable salt formation and low enough to avoid excessive hydrophilicity, which may affect the physical properties of the cements (8). Likewise, other studies have reported widely varying performances of self-adhesive cements, not only regarding bond strength to dentin but also shrinkage behavior (17), physical properties (18), pH values and film thickness (19), water sorption and solubility (20,21). Such cited properties could explain variability in adhesive performance (16).

In the present study G-Cem and RelyX Unicem exhibited a similar performance with higher bond strength results than the other self-adhesive resin cements tested, in accordance with other reports (11,13,16,22). In contrast, Mazzitelli *et al.* (23) found higher bond strength values for RelyX Unicem than for G-Cem. In their study, prematurely fractured specimens were included in the statistical analysis and G-Cem exhibited a double percentage of pre-test failures compared to RelyX Unicem. According to our results, G-Cem also presented more premature failures than RelyX Unicem but they were not included in the statistical analysis and, in any case, were much less than those reported by Mazzitelli *et al.* (23). It may be that the presence of simulated pulpal pressure had contributed to these differences as the same authors had previously stated.

The adhesion mechanism of RelyX Unicem appears to be more chemical than micromechanical in nature (24) as it interacts superficially with the smear layer covered dentin surface and there is no significant infiltration of more than a micrometer into it (25). This is based in the presence of phosphate groups of the functionalized monomers in its composition that effectively bind with calcium in the hydroxyapatite to form a stabilizing attachment between the methacrylated network and the tooth (24). Also, G-Cem contains phosphoric acid esters and 4-META, which in presence of water contained in the cement, are hydrolyzed to form 4-MET. The obtained monomer has also been reported to be capable of a chemical reaction with hydroxyapatite (26).

According to manufacturer’s information, Maxcem Elite also contains an acid monomer, glycerol dimethacrylate dihydrogen phosphate (GPDM), which is partly responsible for the effect of etching and adhesion to the dental structure. However, the indirect composite restorations luted with Maxcem Elite exhibited 86% pre-test failures. A relatively poor bonding ability to dentin had been reported for Maxcem, the previous version of Maxcem Elite, when it was used for luting composite (27), ceramic (22) or zirconia (28), as well as the occurrence of many pre-test failures (13,22). This circumstance was also observed for Smartcem2 that exhibited 88.6% premature failures and the lowest microtensile bond strength mean values in agreement with Viotti *et al.* (13).

Besides a lesser bonding ability, a deficient polymerization has been reported for Maxcem Elite and Smartcem2 in comparison with RelyX Unicem or G-Cem (21). It has been shown that Maxcem and Smartcem2 do not have a relevant acid-base reaction while setting, as do other self-adhesive cements like RelyX Unicem, maintaining a low pH for a long time (19), which could adversely influence the adhesion to dentin and the formation of an optimal cross-linked polymer network.

Regarding Speedcem, despite a lower pre-test failures incidence when compared with Maxcem Elite or Smartcem2, microtensile bond strength values were still significantly lower than those obtained with RelyX Unicem or G-Cem. In a previous paper, the bond strength values to dentin of Speedcem were lower compared to those of RelyX Unicem but similar to those obtained with G-Cem (16).

Finally, it is worth mentioning that microtensile bond strength values reported in the present study were obtained after bonding indirect resin composite restorations to a flat coronal dentin surface, therefore, the influence of unfavorable cavity configuration designs has not been taken into consideration and lower values could be expected.

## Conclusions

The silane and adhesive application after indirect resin composite sandblasting did not improve the bond strength of dentin-composite overlay complex. Selection of the resin cement seems to be a more relevant factor when bonding indirect composites to dentin than its surface treatment.
